# Advances in the pathogenesis of Alzheimer’s disease: a re-evaluation of amyloid cascade hypothesis

**DOI:** 10.1186/2047-9158-1-18

**Published:** 2012-09-21

**Authors:** Suzhen Dong, Yale Duan, Yinghe Hu, Zheng Zhao

**Affiliations:** 1 Shanghai Engineering Research Center for Molecular Therapeutics and New Drug Development, East China Normal University, Shanghai, 200062, China; 2 Institute of Chemical and Translational Genomics, East China Normal University, Shanghai, 200062, China; 3 Key Laboratory of Brain Functional Genomics, Ministry of Education, Shanghai Key Laboratory of Brain Functional Genomics, East China Normal University, 3663 Zhongshan Road (N), Shanghai, 200062, China

**Keywords:** Alzheimer’s disease, A-beta, APP, BACE1, Presenilins, ApoE, Neprilysin/insulin-degrading enzyme

## Abstract

Alzheimer’s disease (AD) is a common neurodegenerative disease characterized clinically by progressive deterioration of memory, and pathologically by histopathological changes including extracellular deposits of amyloid-beta (A-beta) peptides forming senile plaques (SP) and the intracellular neurofibrillary tangles (NFT) of hyperphosphorylated tau in the brain. This review focused on the new developments of amyloid cascade hypothesis with details on the production, metabolism and clearance of A-beta, and the key roles of some important A-beta-related genes in the pathological processes of AD. The most recent research advances in genetics, neuropathology and pathogenesis of the disease were also discussed.

## Review

### Introduction

Alzheimer’s disease (AD) was originally described by Alois Alzheimer in 1906 and was renamed several years later by Emil Kraepelin [1]. AD is characterized clinically by progressive deterioration of memory, and pathologically by histopathological changes including extracellular deposits of amyloid-β (Aβ) peptides forming senile plaques (SP) and the intracellular neurofibrillary tangles (NFT) of hyperphosphorylated tau in the brain, which are commonly regarded as the hallmarks of the disease.

Epidemiological studies have shown that AD is the leading cause of dementia, accounting for about 50% of all cases worldwide [2]. Aging is the most obvious risk factor for developing AD. It was estimated that the age-associated prevalence rate of AD would be doubled every 5 years in the patients beyond 65 years of age [3]. In addition to aging, several other possible biological (such as genetic alterations and polymorphisms, and abnormal immune or inflammatory responses) and environmental factors (such as education, traumatic injury, oxidative stress, drugs, and hormone replacement) and the interactions among these factors have been considered to be contributors to a common pathway leading to AD [4,5].

**Figure 1:**
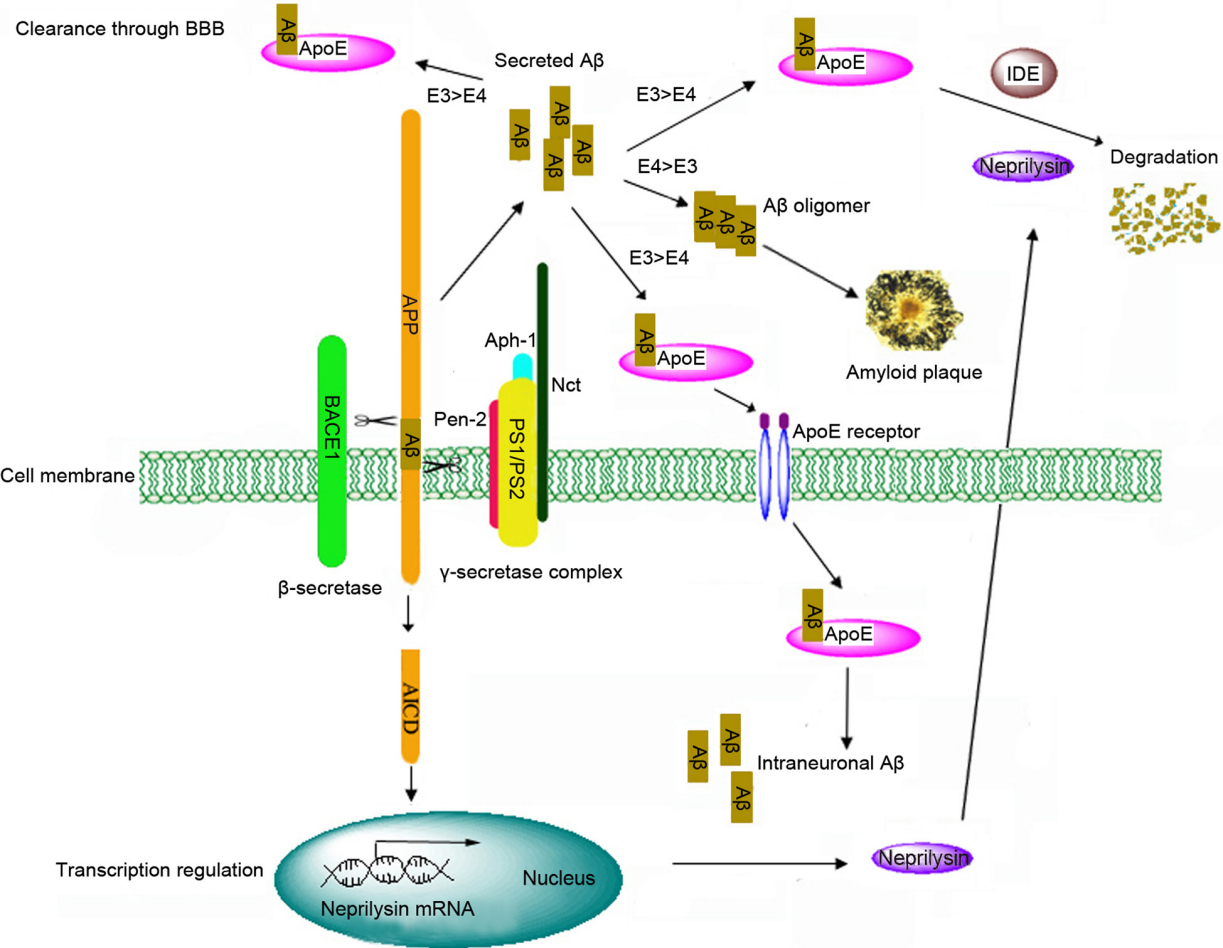
**Aβ and Aβ-related genes in AD.** Aβ is produced by sequential cleavage of APP by β-secretase (BACE1) and γ-secretase. γ-secretase is a multi-protein complex, of which PS1 or PS2 is the catalytic core. After being produced, Aβ is secreted outside the cell and binds to various isoforms of ApoE. These Aβ-binding ApoE isoforms will allow Aβ to undergo metabolism in different pathways, e.g., clearance via BBB, degradation by Aβ-degradating enzymes (IDE or neprilysin), deposition or trafficking into the cell. The affinity of ApoE4 to Aβ is lower than that of ApoE2 or ApoE3. While ApoE2 and ApoE3 help Aβ to be cleared by transport or degradation, ApoE4 mainly induce Aβ to aggregation, implicating it to be a high risk factor for AD. There is a feedback existing in vivo to keep proper Aβ levels. When Aβ is generated, AICD is released, which is translocated into the nucleus and initiates the transcription of neprilysin. Increased neprilysin protein will degradate Aβ and hereby reduces Aβ to a proper level.

Despite the remarkable improvements in our understanding of the pathogenesis of the disease have been made over last several decades, the accurate mechanism of AD remains unclear. Several independent hypotheses have been proposed to address the pathological lesions and neuronal cytopathology in connection with apolipoprotein E (ApoE) genotyping, hyperphosphorylation of cytoskeletal proteins, oxidative stress, abnormal cell cycle re-entry, inflammation and Aβ metabolism. The amyloid metabolic cascade and the posttranslational modification of tau protein are considered to be the most important hypotheses in AD, although none of them or other theories alone is sufficient to explain the diversity of biochemical and pathological abnormalities of AD, which is believed to involve a multitude of cellular and biochemical changes [3]. According to amyloid cascade hypothesis [6,7], accumulation of extracellular senile plaques made primarily by deposits of Aβ peptide is thought to be one of the most prominent pathogenenic mechanisms of AD. Although the direct causal link between Aβ and impaired neuronal function and memory is still under elucidation, it is undoubted that Aβ plays a critical role in the neuropathology of AD. This review focuses on the new developments of amyloid cascade hypothesis and its relevance to the most recent research advances in the genetics, neuropathology and pathogenesis of AD. In the following sections, the recent progress of the studies on genes (see Figure 1) identified to be involved in the production, deposition and degradation of Aβ, the possible contributions of different Aβ assemblies to AD, and their pathological functions are reviewed.

## Aβ-related genes

### Amyloid precursor protein (APP)

APP is an integral membrane glycoprotein expressed in the brain and central nervous system (CNS). It can undergo sequential proteolytic processing by two pathways: the α pathway and the β pathway. In most cases, APP is sequentially cleaved via α pathway by α-secretase and γ-secretase. The α-secretase cleavage of APP is non-amyloidogentic, whereas the β pathway leads to Aβ generation. In the β pathway APP is initially cleaved by β-secretase to release sAPPβ into extracellular space and leave the 99-amino-acids C-terminal fragment (C99) within the membrane. C99 is subsequently processed to 38-43 amino acids by γ-secretase to release Aβ and APP intracellular C-terminal domain (AICD) [8]. In most cases, the γ-cleavage produces Aβ40, while it could also generate a more toxic variant, Aβ42. It has been recently found that γ-secretase activity for Aβ production could be also negatively regulated by α-secretase, indicating a cross-talk between the α pathway and the β pathway [9].

#### Physiological functions

Although APP has been implicated in the pathology of AD, much evidence shows that APP has its own physiological functions, especially in regulation of synaptic function and neuronal activity. Mice lacking APP and APP-like protein 2 show deficits in structure and function in neuromuscular synapses [10]. In cultured hippocampal neurons, lack of APP also affects synapse formation and transmission [11]. On the contrary, mice overexpressing APP exhibit enhanced synaptic plasticity and spatial memory [12]. Kamenetz *et al* found that APP processing could have a normal negative feed-back function in modulating Aβ levels to maintain proper neuronal activity [13]. In addition, APP processing also regulates cholesterol metabolism. When Aβ is produced, AICD is stabilized by Fe65, localized to the nucleus and binds to transcription factor Tip60. The protein-protein interaction initiates the transcription of the Aβ degradation enzyme, neprilysin, thus reduces the Aβ levels [14]. AICD-Fe65-Tip60 complex has been shown to suppress the transcription of lipoprotein receptor LRP1, which is known to regulate ApoE and cholesterol levels in CNS, suggesting a biological interaction between APP and ApoE/cholesterol metabolisms [15]. Furthermore, APP possesses the biological function in controlling cholesterol biosynthesis and sphingomyelin production via Aβ-dependent modulation of neuronal levels of Hydroxymethylglutaryl-CoA reductase (HMGR) and sphingomyelinases (SMases), indicating a functional basis of APP processing for the link between lipids and AD [16]. Endogenous AICD in primary neurons is temporally up-regulated during neuronal differentiation, and such a physiological function is negatively mediated by neuron-specific c-Jun N-terminal kinase JNK3 via phosphorylation of APP [17]. APP and its mammalian paralogs, the amyloid precursor-like proteins 1 and 2, have been demonstrated to be capable of forming homo- and hetero-complexes that exhibit physiological function in promoting trans-cellular adhesion in vivo [18]. Han *et al* also characterized a neuroprotective function of APP in preventing tau hyperphosphorylation via suppressing overactivation of Cdk5 (Cyclin-dependent kinase 5) [19].

#### Pathological functions

It is well known that the pathologcial function of APP lies on its amyloidogenic processing. It has been recognized that many APP mutations cause autosomal dominant early-onset AD. Increasing of gene copy number including genomic duplication in the APP locus [20,21] may also lead to AD dementia in earlier life. Interestingly, a recently identified mutation adjacent to β-site (A673T) of APP gene was shown to result in Aβ reduction and protection against cognitive decline in the elderly without AD [22]. On the other hand, however, overexpression of FAD-linked mutant APP could lead to olfactory sensory neuron apoptosis in the absence of amyloid plaque, which might be the mechanism of deficits in odor detection, one of the earliest AD symptoms [23]. All these indicate that both APP genomic duplication and mutations can lead to changes in APP function and subsequent Aβ metabolism, strongly implicating a central role of not only APP but also its β-cleavage in pathogenesis of AD. To identify the pathological functions of APP, many APP transgenic mice including wild-type human APP and FAD-linked APP mutations have been generated. FAD-linked APP mutation mice show an increase in the amount, length, and fibrillogenic generation of Aβ species and have amyloid deposits at the age of 18 months [24] while, surprisingly, mice overexpressing APP do not develop AD pathologies or memory deficits but instead exhibit enhanced spatial memory, which depends on the function of AICD generated by β-secretase-mediated cleavage [12]. Studies on APP mutation transgenic mice have given us much information of AD pathogenesis, but the molecular mechanisms still need further investigation.

### Beta-site APP cleaving enzyme 1 (BACE1)

BACE1 is known as the major β-secretase to cleave APP at β-site to produce β-CTF for Aβ generation in neurons [25]. BACE1 and its homolog, BACE2, have different transcriptional regulations and functions. BACE1 knockout mice are almost normal without Aβ generation [26], and BACE1 deficits can rescue the memory impairment and cholinergic dysfunction in mutant human APP transgenic mice [27]. Repetto *et al* demonstrated that overexpression of BACE1 in H4 human cells can regulate APP intracellular signaling by interaction with the ShcA adaptor protein [28]. BACE1-mediated β-cleavage has been showed to be physiologically modulated by different spliced transcripts [29] and the activation of protein kinase C [30]. Impaired intracellular calcium homeostasis may stimulate BACE1 gene expression via nuclear factor of activated T cells 1 (NFAT 1) signaling pathway, leading to accelerated production of Aβ [31]. BACE1 could be modulated by Aβ42, but not Aβ40, via an NFκB-dependent signaling pathway [32], and Aβ42-positive plaques could increase BACE1 levels in surrounding neurons before neuron loss occurs [33]. Aβ42 could also induce expression of BACE1-antisense transcript, a natural regulator of BACE1 expression, which increased BACE1 mRNA stability [34]. Thus, increased BACE1 levels might be a positive feedback for Aβ42 to initiate the amyloidogenesis of AD. In addition, BACE1-dependent cleavage of low density lipoprotein receptor-related protein (LRP) can mediate the endocytosis of APP and ApoE [35] and has been suggested to be involved in the pathology of AD.

### Presenilin (PS) 1 and 2

PS is an eight membrane-spanning protein with an N-terminus, a ‘loop’ domain between transmembrane (TM) domain six and seven, and a C-terminus that is oriented toward the cytoplasm. Aspartate residues at position D275 (in TM6) and D385 (in TM7) are critical for PS function [36]. There are two PS genes: PS1 and PS2. PS, nicastrin, aph-1, and pen-2 form the active γ-secretase complex while PS is the catalytic core of the complex [37]. γ-secretase cleaves not only APP but many other type I transmembrane proteins (such as Notch, cadherins and LRP) as well [38], strongly implicating PS in both AD pathogenesis and many other neuronal physiological activities including development, calcium homeostasis and apoptosis.

#### PS gain of function

PS1 mutations are the most common genetic cause for early-onset familial AD (FAD). PS genes harbor about 90% of identified FAD mutations. There have been more than 100 PS1 mutations being described. Some of them, such as L85P, P117L, P117S, insF1, and L166P, are associated with very early onset (usually before age of 30 years old) of cognitive decline [39]. Many of the PS1 mutations lead to an increase in relative production of more toxic Aβ42 peptides. The prevailing amyloid hypothesis posits that deposits of Aβ peptides, especially the more hydrophobic and aggregation-prone Aβ42, initiates a pathogenic cascade, leading to neurodegeneration in AD [40]. This has been referred to as the toxic gains-of-function of PS in triggering neurodegeneration in AD. The amyloid cascade hypothesis is supported by the results from FAD-linked mutant transgenic mice. PS1 mutation significantly accelerates the rate of Aβ deposition in mutant APP transgenic mice [24]. Expression of human mutant PS1 in PS1 null mice is sufficient to elevate Aβ1-42, supporting a gain-of-function activity of PS1 mutation [41].

#### PS loss of function

However, the most recent evidence from several independent PS transgenic model-based studies emerged that supports the “PS loss of function” hypothesis as a potential pathogenic mechanism of AD. Firstly, mice lacking both PSs in the forebrain show AD-like progressive neurodegenerative phenotypes including forebrain degeneration, impaired synaptic plasticity and spatial memory without Aβ production [42-46]. A number of PS1 mutations (L113P, G183V and insR352) have been found in patients with familial forms of frontotemporal dementia (FTD), a common neurodegenerative dementia that lacks amyloidogenesis [47-49]. These observations suggest that neurodegeneration can take place in the absence of Aβ.

PS genes have been identified to play an important role in many normal physiological activities. These physiological functions can be classified to γ-secretase-dependent and -independent functions. There are many identified γ-secretase substrates. By cleavage of these substrates, PSs mediate their multiple functions in development, calcium homeostasis, cell adhesion, transport, trafficking/localization, and apoptosis [36,38,50]. FAD-linked PS mutations might impair the γ-secretase-dependent proteolysis of some of the substrates, such as Notch, N-cadherin and tyrosinase, resulting in loss of the related functions of PS [51,52]. Meanwhile, FAD-linked PS mutations might also impair some γ-secretase-independent functions, such as the regulation of β-catenin-dependent signaling [53], modulation of phosphatidylinositol 4,5-bisphosphate metabolism [54], endoplasmic reticulum [Ca^2+^ leak function [55], PI3K/Akt signaling pathway-dependent neuroprotective roles [56], synaptic homeostasis [57] and fast axonal transport of APP [58]. In addition to involving in Aβ generation, PS genes participate in the regulation of Aβ degradation mediated by AICD-dependent transcriptional modulation of the degrading enzyme, neprilysin [14]. FAD-linked PS mutations may disrupt the physiological function of PSs in regulating Aβ levels.

Surprisingly, many γ-secretase inhibitors at low concentration enhance Aβ42 production while reducing Aβ40 levels, similar to the effects of FAD-linked PS mutations [59-61], suggesting that PS mutation could result in a partial loss of its function. Furthermore, PS mutations are scattered throughout the protein’s N-terminus, C-terminus and transmembrane domains, occurring at about 20% of the amino acid residues. As it is impossible for different PS mutations to gain the same toxic function, it is therefore most likely that the loss of normal PS function by ‘random’ changes of amino acid residues is the culprit for triggering AD pathogenesis. However, the “PS loss of function” hypothesis is still unable to explain the exact mechanism for FAD-linked APP mutations that cause AD. In this regard, it has been assumed that Aβ42 might act as an inhibitor of γ-secretase. APP mutations may interfere with the physiological roles of PS and hereby initiate the pathogenic cascades of AD [51]. Further investigations are required to confirm the hypothesis.

### Apolipoprotein E (ApoE) and other apolipoproteins

Apolipoproteins play important roles in regulating Aβ pathology. ApoE is the predominant apolipoprotein in the CNS and is synthesized and secreted mainly by astrocytes and microglias [62,63]. ApoE has critical roles in transporting lipids among CNS cells to keep lipid homeostasis, repairing injured neurons, maintaining synaptic connections, and scavenging toxins. ApoE gene encodes three alleles: ApoE2, ApoE3 and ApoE4. The alleles differ only in two residues at sites 112 and 158. ApoE3 has Cys-112 and Arg-158, ApoE4 has arginine and ApoE2 cysteine at both sites. The differences between the alleles determine their distinct functions. ApoE2 is neuroprotective while ApoE4 is related to a variety of diseases.

It has been recognized that ApoE4 is the major genetic risk factor for sporadic AD. ApoE4 is associated with cognitive deficits [64], and the effect of ApoE4 is moderated by cholesterol levels [65]. In contrast to ApoE2 and ApoE3, ApoE4 is more sensitive to stress or injury, which causes neuron-specific proteolysis with the formation of a bioactive toxic C-terminal fragment [66]. Transgenic mice expressing high levels of carboxyl terminal-cleaved product ApoE4 (272–299) in the brain die 2–4 months after birth. The cortex and hippocampus of the transgenic mice display AD-like neurodegenerative alterations [67].

ApoE acts as the Aβ chaperone and binds to different forms of Aβ, leading to changes in the structure, toxicity and deposition of Aβ [68,69]. Pharmacological blocking ApoE-Aβ interaction can significantly reduce the formation of amyloid plaques and attenuate the deficits of memory in the transgenic mice carrying a Swedish K670L/M671L APP mutation (APP_SWE_) or a K670L/M671L APP plus a PS1 M146L mutation (APP_SWE_/PS1) [70]. The effects of ApoE on Aβ depositions are supported by the observation that intake of sugar-sweetened water induces amyloidosis and memory impairment and increases ApoE levels in the brain of a transgenic mouse model of AD [71]. Besides, it is recently demonstrated that increased expression of ApoE by the retinoid X receptors agonist results in enhanced clearance of soluble Aβ and reduced Aβ plaque, and leads to reversal of cognitive deficits and improvement of synaptic functions in an AD mouse model [72]. Nevertheless, increasing evidence suggests that the regulatory effect of ApoE on Aβ deposition appears to be isoform-specific (ApoE4 > ApoE3 > ApoE2) and gene dosage-related [73]. ApoE promotes the proteolytic degradation of Aβ by modulating the activity of Aβ degrading enzyme, which depends on ApoE isoform structure and the lipidation status [69,74]. ApoE also participates in the regulation of Aβ production through LRP pathway [75]. Given that APP processing is dependent on membrane cholesterol levels and that ApoE is the transporter of cholesterol [76], ApoE might therefore be a important player in Aβ generation. In fact, it has been reported that activation of the amyloid cascade may isoform-specifically induce lysosomal activation and neurodegeneration of hippocampal CA1, entorhinal and septal neurons, which are responsible for the marked cognitive deficits in apolipoprotein transgenic mice [77]. ApoE4-induced impairments of neuroplasticity following environmental stimulation are also found to be mediated by intraneuronal oligomerized Aβ [78]. Furthermore, C-terminal fragment of ApoE could induce tau phosphorylation in neurons that represents another character in AD brain, depending on both the isoform and cellular source of ApoE [67,79].

In addition to ApoE, other apolipoproteins, such as ApoA-IV, were also found to regulate Aβ metabolism. Genetic ablation of ApoA-IV in an AD mouse model accelerates Aβ deposition, neuron loss and cognitive impairment [80].

### Neprilysin/insulin-degrading enzyme

While familial early-onset AD is associated with increased Aβ production, defective Aβ degradation may be involved in late-onset AD (LOAD), which constitutes approximately 90% of all AD cases [81]. Many enzymes including, but not limited to, neprilysin (NEP) and insulin-degrading enzyme (IDE) have been implicated for a role in degrading Aβ [82]. NEP and IDE are reduced in AD, and increasing evidence indicates an involvement of them in the imbalance of Aβ production and clearance relating to AD pathology.

#### Neprilysin (NEP)

NEP is a 90 ~ 110 kDa plasma membrane glycoprotein of the neutral zinc metalloendopeptidase family that degrades enkephalins, endothelins, and Aβ peptides [82]. Particularly, NEP is the major enzyme to degrade soluble extracellular Aβ in the brain. Recent studies demonstrated that NEP levels decline in an age-dependent manner and inversely correlate with levels of insoluble Aβ in the temporal and frontal cortex of AD and normal brain [83,84]. NEP expression or activities are decreased significantly in AD brain [85,86]. The finding that possession of ApoE4 was related to obvious reduction in NEP levels [85] suggests that down-regulation of NEP might be implicated in AD pathogenesis.

The transcription of NEP has been demonstrated to be regulated by AICD which is released during the Aβ generation [14]. There is a physiological negative feedback in vivo that keeps Aβ homeostasis, in which Aβ production could lead to translocation of AICD to nucleus and transactivation of NEP. NEP therefore has a role in governing the balance between Aβ production and degradation. If such a balance is disrupted, Aβ would come to be oligomerized and lead to formation of the fibrillar Aβ protein (fAβ). The resulting fAβ can inhibit the proteolytic activities of the proteases by binding to NEP and IDE [87], leading to the formation of a positive feedback that accelerates amyloid deposition. Indeed, studies on transgenic mice of AD have shown that NEP-mediated degradation of Aβ plays a key role in AD neurodegeneration and serves as a novel therapeutic approach to AD [88]. These findings also suggest that AD pathogenesis might result from deficits in Aβ clearance. On the other hand, however, recent observations in drosophila demonstrated that NEP overespression could result in the inhibition of CREB-mediated transcription, age-dependent axon degeneration and shortened lifespan [89]. Studies on crossing hAPP transgenic mice and NEP transgenic mice also showed that, although NEP overexpression inhibits plaque formation, it fails to reduce pathogenic Aβ oligomers and improve the impaired learning and memory function [90].

#### Insulin-degrading enzyme (IDE)

IDE is an 110 kDa zinc metalloendopeptidase that highly expresses in the liver, testis, muscle and brain [82]. The enzyme has been implicated in the pathogenesis of AD and type II diabetes due to its capabilities in degrading Aβ, AICD [91,92], amylin, insulin and insulin-like growth factors [82]. IDE gene is located in chromosome 10 that is highly associated with later-onset AD (LOAD) [93]. Some genetic variants of IDE have also been strongly implicated in LOAD [94,95]. IDE mRNA and protein levels are markedly decreased in hippocampus of AD patients with ApoE ε4 allele, the genotyping known as a high risk factor for LOAD [96]. Membrane-bound IDE levels and its activity are significantly decreased in subjects with mild cognitive impairment (MCI) and appear to decrease continuously during the conversion from MCI to AD [97]. IDE activity is reduced in affected versus unaffected subjects of three chromosome 10-linked AD pedigrees, although no significant difference of IDE expression has been observed [98]. However, recent studies on transgenic AD mouse models showed that cortical IDE mRNA and protein levels are elevated in parallel with Aβ40 and Aβ42 generation [99]. In transgenic tg2576 mice, IDE expression is increased with age and is located around amyloid plaque as a result of Aβ-induced inflammation [100]. This phenomena is similar to the observation that IDE is immunopositive in senile plaques in human AD brain [101]. Studies on triple-transgenic mice (hAPPswe/PS1 M146V/hTau P301L) showed that the expression of IDE was regulated by 17beta-estrodiol via an ERbeta/PI3K pathway [102]. Unlike NEP that hydrolyzes both monomeric and oligomeric Aβ, IDE is found to degrade only soluble monomeric Aβ [103]. A recent study by Llovera *et al* demonstrated that the catalytic domain of IDE could form a stable complex with Aβ, which might disrupt Aβ clearance and facilitate AD neurodegeneration [104]. There have been also studies showing that IDE can cleave C-terminal domain of human acetylcholinesterase (hAChE) and trigger its conformational conversion from α to β-structure, which acts as the seed of Aβ fibrils and enhances the rate of amyloid elongation [105]. This suggests an important role of IDE digestion of C-terminal domain of hAChE in amyloidogenic pathogenesis of AD.

IDE plays essential role in insulin homeostasis, implicating a close relationship between AD and type II diabetes (DM2). A large body of evidence has indicated that cognitive capacity is often impaired in patients with diabetes [106] while insulin resistance is a high risk factor of AD [107]. IDE knockout mice exhibit hallmarks of both AD and DM2 [92]. Diet-induced insulin resistance leads to increased γ-secretase activity and decreased IDE activity, resulting in elevated Aβ40 and Aβ42 levels in the brain of Tg2576 mice [108]. Further exploration of the underlying mechanism has shown that defective insulin receptor signaling may lead to up-regulation of Aβ generation. Insulin resistance induced by intake of sucrose-sweetened water or a safflower oil-enriched diet exacerbates the AD pathology in transgenic AD animal models [71,109].

### Aβ and neurodegeneration

Although it has been widely accepted that Aβ plays a central role in the onset and progression of AD pathology, it remains unclear whether soluble or insoluble Aβ located in extracellular or intracellular is the culprit to impaired neuronal function and memory. Meanwhile, much progress based on neurotoxic lesion, pharmacological, genetic, and neurophysiological studies in recent years has led to identification of many new physiological and biological alterations, such as mitochondrial dysfunction, oxidative stress, synaptic transmission, axonal trafficking and membrane disruption, that are responsible for the functions of Aβ with significant implications in developing AD [110,111]. In the following sub-sections, we selectively review the present research status in characterization of neurotoxic form of Aβ, and in the pathological functions of Aβ in synaptic dysfunction and neuronal inflammation.

### Aβ: soluble or insoluble, extracellular or intracellular, which one is neurotoxic form to AD?

In the last two decades, Aβ hypothesis has been the focus of AD researches. According to the hypothesis, deposition of Aβ peptide is the primary cause of driving AD degeneration and all of the other pathological features including intracellular neurofibrillary tangles (NFT) and neuron loss are the downstream events of the amyloid cascade [40]. The hypothesis, however, has been challenged in recent years [112]. Appearance of large “cotton wool” plaques resulting from PS1 mutations has been demonstrated to be associated with some special symptoms such as spastic paraparesis rather than early-onset AD [113]. Decreased dendritic spine density, impaired synaptic plasticity, and cognitive dysfunction occur long before amyloid depositions that appear at 18 months in Tg2576 mice [114]. Hippocampal neuron loss in AD mouse models has been observed both at the site of amyloid aggregation and in areas distant from plaques [115].

The classical view is that Aβ is deposited extracellularly, however, emerging evidence from transgenic mice and human patients has indicated that this peptide can also be accumulated intraneuronally that contributes to AD pathogenesis [116]. A PS1 mutated transgenic mouse model with intracellular Aβ accumulation but without amyloid plaques exhibits AD-like neurodegeneration [117]. Results from the triple-transgenic AD mouse model (hAPPswe/PS1 M146V/hTau P301L) showed that impaired synaptic plasticity and cognitive dysfunction occur prior to the apparent plaques, and are correlated with the accumulation of intraneuronal Aβ in hippocampus and amygdale [118,119]. Intracellular Aβ has been found to accumulate before the generation of amyloid plaques in many other AD mouse models [120-124] and in human AD [20,21,125]. As mentioned above, overexpression of FAD-linked mutant APP alone could induce the apoptosis of olfactory sensory neuron and this neurodegeneration is reversible, suggesting that amyloid plaques are not necessary for AD neurodegeneration [23].

Evidence has also emerged that the soluble Aβ, but not amyloid plaques, initiates pathological cascade. Aβ dimmers derived from untreated human cerebrospinal fluid (CSF) suppress hippocampal synaptic plasticity in vivo [126]. Neuron exposure to prefibrillar Aβ can cause tau-dependent microtubule disassembly [127]. Aβ oligomers have been observed to disrupt calcium signaling [128], affect the function of NMDA receptor [129,130], and induce oxidative stress [131] and mitochondrial dysfunction [132]. Furthermore, in vitro studies have demonstrated that β-sheet intermediate (Iβ) of Aβ prior to fibril formation is more toxic than the fibrils [133]. It has also been shown that soluble Aβ oligomers induce reduction in postsynaptic receptors and disruptions of synaptic morphology in cultured hippocampal neurons [134,135]. Notably, intracerebroventricular injection of AD brain-derived extracts containing soluble Aβ could lead to obvious inhibition of hippocampal LTP in rats, supporting the role of SDS-stable Aβ dimer in mediating synaptic plasticity disruption [136].

Pyroglutamate-amyloid- β (pE3- Aβ), an N-terminal truncated Aβ species, has recently been found in Aβ deposits specific to AD brain but absent in normal aging. Transgenic mice expressing this kind of truncated Aβ showed progressive neurodegeneration including neuron loss, impaired LTP, microglia activation and astrocytosis [137]. A signal transduction pathway of soluble Aβ oligomer has recently been delineated, in which oligomeric Aβ activates Fyn kinase by binding to cellular prion protein (PrPc) and results in the phosphorylation of NR2B subunit of NMDA receptor, and eventually leads to dendritic spine loss and altered synaptic function [138].

In contrast to the observations mentioned above, Lesne *et al* identified the extracellular accumulation of a soluble 56-kDa Aβ assembly (termed Aβ*56) composed of 12 Aβ peptides that contributes to the memory impairment in Tg2576 mice [139]. This finding has been supported by others using transgenic mice with increased formation of amyloid plaques but reduced Aβ*56 levels [140]. Taken together, although increasing data have been accumulated that strongly suggest the soluble Aβ and intracellular Aβ to be more suspicious in underlying AD pathogenesis, the involvement of extracellular Aβ in pathologies of the disease is still not neglectable.

### Other pathological functions of Aβ

#### Aβ and synaptic dysfunction

Aβ has long been shown to affect excitatory synaptic neurotransmission [13] and hippocampal synaptic plasticity [110,126,141]. Aβ oligomers are able to bind specifically to excitatory pyramidal neurons and affect their synaptic structure, composition and density and the membrane expression of NMDA receptor [135]. Similar observations on rat hippocampal slices have also shown that Aβ oligomers induce loss of hippocampal synapses and spines in a NMDA receptor-dependent manner [130]. Aβ exhibits a specific inhibitory role in a presynaptic P/Q calcium current, which is required for synaptic plasticity [128]. Aβ also plays an important role in activity-dependent presynaptic vesicle release [142]. Moreover, Aβ can induce neuronal network dysfunction including abnormal induction of excitatory neuronal activity and compensatory inhibitory circuits [143]. The abnormalities of synapse and neuronal network resulting from Aβ might be the physiological basis of cognitive decline in AD animal models and patients.

#### Aβ and inflammation

Microglia is rapidly recruited around amyloid plaques after its appearance [144]. Aβ can trigger the translocation of microglia from bone marrow to the sites around amyloid plaques [145]. Aβ up-regulates P38 MAPK or p44/42 MAPK signaling, which may lead to microglia activation with release of cytokines including tumor necrosis factor α (TNF-α) and interleukin-1 β (IL1-β) [146]. The microglia around plaques maintains the stability of the plaques [147]. Both pharmacological blockade and genetic knock-out of TNF-α or iNOS down-regulate Aβ-induced cognitive dysfunction in AD mouse model, revealing that TNF-α and iNOS are key mediator of Aβ neurotoxicity [148]. Genetic disruption of transforming growth factor β (TGF-β) signaling mitigates Aβ levels and amyloid plaques, and partially rescues the cognitive abnormality in Tg2576 mice [149]. However, the roles of TGF-β signaling are in a debate. Tesseur I *et al* showed that deficiency in TGF-β signaling promotes Aβ accumulation and neuronal degeneration [150]. Accumulating evidence also suggests that functional loss of TGF-β signaling may contribute to Aβ-induced neurodegeneration and tau pathology, indicating a neuroprotective role of this pathway [151].

## Conclusion

AD is a complex neurodegenerative disease involving the interactions among various potential biological and environmental factors. Among them, abnormal processes of Aβ production, degradation and deposition have been strongly implicated in the underlying neuropathology and neuropathogenesis of familial earlier-onset and sporadic later-onset forms of AD. Genes involved in these processes, including APP, BACE1, PS1/2, ApoE, NEP, IDE and so on, play important roles in AD initiation and progression. Further dissection with depth and breadth of genetic influences may help defining the precise mechanisms involved in the disease pathogenesis, and eventually leading to development of new arrays of therapeutics with symptomatic effects or disease-modifying potential.

## Abbreviations

AD: Alzheimer’s disease; Aβ: Amyloid-β; SP: Senile plaques; NFT: Neurofibrillary tangles; ApoE: Apolipoprotein E; CNS: Central nervous system; APP: Amyloid precursor protein; C99: C-terminal fragment; AICD: APP intracellular C-terminal domain; LRP1: Lipoprotein receptor-related protein; HMGR: Hydroxymethylglutaryl-CoA reductase; SMases: Sphingomyelinases; JNK: c-Jun N-terminal kinase; Cdk5: Cyclin-dependent kinase 5; FAD: Familiar Alzheimer’s disease; BACE1: Beta-site APP cleaving enzyme 1; β-CTF: β-C-terminal fragment; BACE2: Beta-site APP cleaving enzyme 2; NFAT 1: Nuclear factor of activated T cells 1; NRG1: Neuregulin 1; PS: Presenilin; TM: Transmembrane; aph-1: Anterior pharynx-defective 1; pen-2: Presenilin enhancer 2; FTD: Frontotemporal dementia; PI3K: Phosphoinositide_3-kinase; Akt: Protein kinase B; NEP: Neprilysin; IDE: Insulin-degrading enzyme; ECEs: Endothelin-converting enzymes; ACE: Angiotensin-converting enzyme; MMPs: Plasmin and matrix metalloproteinases; LOAD: Later-onset AD; MCI: Mild cognitive impairment; ERbeta: Estrogen receptor beta; hAChE: Human acetylcholinesterase; DM2: Type II diabetes; PrPc: Cellular prion protein; MAPK: Mitogen-activated protein kinase; TNF-α: Tumor necrosis factor α.; IL1-β: Interleukin-1 β; GSK3: Glycogen synthase kinase 3.

## Competing interests

The authors declare that they have no competing interests.

## Authors’ contributions

SZ Dong and YL Duan collected the reference materials and drafted the manuscript. YH Hu and Z Zhao conceived of the study, and participated in its design and coordination and helped to draft the manuscript. All authors read and approved the final manuscript.

## References

[B1] Möller HJ Graeber MB The case described by Alois Alzheimer in 1911 Eur Arch Psychiatry Clin Neurosci 1998 248 111 122 9728729 10.1007/s004060050027

[B2] Mount C Downton C Alzheimer disease: progress or profit? Nat Med 2006 12 780 784 16829947 10.1038/nm0706-780

[B3] Bachman DL Wolf PA Linn RT Knoefel JE Cobb JL Belanger AJ White LR D’Agostino RB Incidence of dementia and probable Alzheimer’s disease in a general population: the Framingham Study Neurology 1993 43 515 519 8450993 10.1212/wnl.43.3_part_1.515

[B4] Hardy J The Alzheimer family of diseases: many etiologies, one pathogenesis? Proc Natl Acad Sci USA 1997 94 2095 2097 9122152 10.1073/pnas.94.6.2095 PMC33655

[B5] Small GW The pathogenesis of Alzheimer’s disease J Clin Psychiatry 1998 59 Suppl 9 7 14 9720481

[B6] Hardy J Alzheimer’s disease: the amyloid cascade hypothesis: an update and reappraisal J Alzheimers Dis 2006 9 151 153 16914853 10.3233/jad-2006-9s317

[B7] Hardy JA Higgins GA Alzheimer’s disease: the amyloid cascade hypothesis Science 1992 256 184 185 1566067 10.1126/science.1566067

[B8] Selkoe DJ Normal and abnormal biology of the beta-amyloid precursor protein Annu Rev Neurosci 1994 17 489 517 8210185 10.1146/annurev.ne.17.030194.002421

[B9] Tian Y Crump CJ Li YM Dual role of alpha-secretase cleavage in the regulation of gamma-secretase activity for amyloid production J Biol Chem 2010 285 32549 32556 20675367 10.1074/jbc.M110.128439 PMC2952257

[B10] Wang P Yang G Mosier DR Chang P Zaidi T Gong YD Zhao NM Dominguez B Lee KF Gan WB Zheng H Defective neuromuscular synapses in mice lacking amyloid precursor protein (APP) and APP-Like protein 2 J Neurosci 2005 25 1219 1225 15689559 10.1523/JNEUROSCI.4660-04.2005 PMC6725967

[B11] Priller C Bauer T Mitteregger G Krebs B Kretzschmar HA Herms J Synapse formation and function is modulated by the amyloid precursor protein J Neurosci 2006 26 7212 7221 16822978 10.1523/JNEUROSCI.1450-06.2006 PMC6673945

[B12] Ma H Lesne S Kotilinek L Steidl-Nichols JV Sherman M Younkin L Younkin S Forster C Sergeant N Delacourte A Involvement of beta-site APP cleaving enzyme 1 (BACE1) in amyloid precursor protein-mediated enhancement of memory and activity-dependent synaptic plasticity Proc Natl Acad Sci USA 2007 104 8167 8172 17470798 10.1073/pnas.0609521104 PMC1859992

[B13] Kamenetz F Tomita T Hsieh H Seabrook G Borchelt D Iwatsubo T Sisodia S Malinow R APP processing and synaptic function Neuron 2003 37 925 937 12670422 10.1016/s0896-6273(03)00124-7

[B14] Pardossi-Piquard R Petit A Kawarai T Sunyach C da Alves Costa C Vincent B Ring S D’Adamio L Shen J Muller U Presenilin-dependent transcriptional control of the Abeta-degrading enzyme neprilysin by intracellular domains of betaAPP and APLP Neuron 2005 46 541 554 15944124 10.1016/j.neuron.2005.04.008

[B15] Liu Q Zerbinatti CV Zhang J Hoe HS Wang B Cole SL Herz J Muglia L Bu G Amyloid precursor protein regulates brain apolipoprotein E and cholesterol metabolism through lipoprotein receptor LRP1 Neuron 2007 56 66 78 17920016 10.1016/j.neuron.2007.08.008 PMC2045076

[B16] Grimm MO Grimm HS Patzold AJ Zinser EG Halonen R Duering M Tschape JA De Strooper B Muller U Shen J Hartmann T Regulation of cholesterol and sphingomyelin metabolism by amyloid-beta and presenilin Nat Cell Biol 2005 7 1118 1123 16227967 10.1038/ncb1313

[B17] Kimberly WT Zheng JB Town T Flavell RA Selkoe DJ Physiological regulation of the beta-amyloid precursor protein signaling domain by c-Jun N-terminal kinase JNK3 during neuronal differentiation J Neurosci 2005 25 5533 5543 15944381 10.1523/JNEUROSCI.4883-04.2005 PMC6724978

[B18] Soba P Eggert S Wagner K Zentgraf H Siehl K Kreger S Lower A Langer A Merdes G Paro R Homo- and heterodimerization of APP family members promotes intercellular adhesion EMBO J 2005 24 3624 3634 16193067 10.1038/sj.emboj.7600824 PMC1276707

[B19] Han P Dou F Li F Zhang X Zhang YW Zheng H Lipton SA Xu H Liao FF Suppression of cyclin-dependent kinase 5 activation by amyloid precursor protein: a novel excitoprotective mechanism involving modulation of tau phosphorylation J Neurosci 2005 25 11542 11552 16354912 10.1523/JNEUROSCI.3831-05.2005 PMC6726015

[B20] Cabrejo L Guyant-Marechal L Laquerriere A Vercelletto M De la Fourniere F Thomas-Anterion C Verny C Letournel F Pasquier F Vital A Phenotype associated with APP duplication in five families Brain 2006 129 2966 2976 16959815 10.1093/brain/awl237

[B21] Rovelet-Lecrux A Hannequin D Raux G Le Meur N Laquerriere A Vital A Dumanchin C Feuillette S Brice A Vercelletto M APP locus duplication causes autosomal dominant early-onset Alzheimer disease with cerebral amyloid angiopathy Nat Genet 2006 38 24 26 16369530 10.1038/ng1718

[B22] Jonsson T Atwal JK Steinberg S Snaedal J Jonsson PV Bjornsson S Stefansson H Sulem P Gudbjartsson D Maloney J A mutation in APP protects against Alzheimer’s disease and age-related cognitive decline Nature 2012 488 96 99 22801501 10.1038/nature11283

[B23] Cheng N Cai H Belluscio L In vivo olfactory model of APP-induced neurodegeneration reveals a reversible cell-autonomous function J Neurosci 2011 31 13699 13704 21957232 10.1523/JNEUROSCI.1714-11.2011 PMC3190161

[B24] Borchelt DR Ratovitski T van Lare J Lee MK Gonzales V Jenkins NA Copeland NG Price DL Sisodia SS Accelerated amyloid deposition in the brains of transgenic mice coexpressing mutant presenilin 1 and amyloid precursor proteins Neuron 1997 19 939 945 9354339 10.1016/s0896-6273(00)80974-5

[B25] Cai H Wang Y McCarthy D Wen H Borchelt DR Price DL Wong PC BACE1 is the major beta-secretase for generation of Abeta peptides by neurons Nat Neurosci 2001 4 233 234 11224536 10.1038/85064

[B26] Luo Y Bolon B Kahn S Bennett BD Babu-Khan S Denis P Fan W Kha H Zhang J Gong Y Mice deficient in BACE1, the Alzheimer’s beta-secretase, have normal phenotype and abolished beta-amyloid generation Nat Neurosci 2001 4 231 232 11224535 10.1038/85059

[B27] Ohno M Sametsky EA Younkin LH Oakley H Younkin SG Citron M Vassar R Disterhoft JF BACE1 deficiency rescues memory deficits and cholinergic dysfunction in a mouse model of Alzheimer’s disease Neuron 2004 41 27 33 14715132 10.1016/s0896-6273(03)00810-9

[B28] Repetto E Russo C Venezia V Nizzari M Nitsch RM Schettini G BACE1 overexpression regulates amyloid precursor protein cleavage and interaction with the ShcA adapter Ann N Y Acad Sci 2004 1030 330 338 15659814 10.1196/annals.1329.041

[B29] Mowrer KR Wolfe MS Promotion of BACE1 mRNA alternative splicing reduces amyloid beta -peptide production J Biol Chem 2008 283 18694 18701 18468996 10.1074/jbc.M801322200

[B30] Wang L Shim H Xie C Cai H Activation of protein kinase C modulates BACE1-mediated beta-secretase activity Neurobiol Aging 2008 29 357 367 17157415 10.1016/j.neurobiolaging.2006.11.001 PMC2278113

[B31] Cho HJ Jin SM Youn HD Huh K Mook-Jung I Disrupted intracellular calcium regulates BACE1 gene expression via nuclear factor of activated T cells 1 (NFAT 1) signaling Aging Cell 2008 7 137 147 18081741 10.1111/j.1474-9726.2007.00360.x

[B32] Buggia-Prevot V Sevalle J Rossner S Checler F NFkappaB-dependent control of BACE1 promoter transactivation by Abeta42 J Biol Chem 2008 283 10037 10047 18263584 10.1074/jbc.M706579200

[B33] Zhao J Fu Y Yasvoina M Shao P Hitt B O’Connor T Logan S Maus E Citron M Berry R Beta-site amyloid precursor protein cleaving enzyme 1 levels become elevated in neurons around amyloid plaques: implications for Alzheimer’s disease pathogenesis J Neurosci 2007 27 3639 3649 17409228 10.1523/JNEUROSCI.4396-06.2007 PMC6672403

[B34] Faghihi MA Modarresi F Khalil AM Wood DE Sahagan BG Morgan TE Finch CE St Laurent G 3rd Kenny PJ Wahlestedt C Expression of a noncoding RNA is elevated in Alzheimer’s disease and drives rapid feed-forward regulation of beta-secretase Nat Med 2008 14 723 730 18587408 10.1038/nm1784 PMC2826895

[B35] Li Y Cam J Bu G Low-density lipoprotein receptor family: endocytosis and signal transduction Mol Neurobiol 2001 23 53 67 11642543 10.1385/MN:23:1:53

[B36] Vetrivel KS Zhang YW Xu H Thinakaran G Pathological and physiological functions of presenilins Mol Neurodegener 2006 1 4 16930451 10.1186/1750-1326-1-4 PMC1513131

[B37] De Strooper B Aph-1, Pen-2, and Nicastrin with Presenilin generate an active gamma-Secretase complex Neuron 2003 38 9 12 12691659 10.1016/s0896-6273(03)00205-8

[B38] Koo EH Kopan R Potential role of presenilin-regulated signaling pathways in sporadic neurodegeneration Nat Med 2004 10 Suppl S26 S33 15272268 10.1038/nm1065

[B39] Larner AJ Doran M Clinical phenotypic heterogeneity of Alzheimer’s disease associated with mutations of the presenilin-1 gene J Neurol 2006 253 139 158 16267640 10.1007/s00415-005-0019-5

[B40] Hardy J Selkoe DJ The amyloid hypothesis of Alzheimer’s disease: progress and problems on the road to therapeutics Science 2002 297 353 356 12130773 10.1126/science.1072994

[B41] Qian S Jiang P Guan XM Singh G Trumbauer ME Yu H Chen HY Van de Ploeg LH Zheng H Mutant human presenilin 1 protects presenilin 1 null mouse against embryonic lethality and elevates Abeta1-42/43 expression Neuron 1998 20 611 617 9539133 10.1016/s0896-6273(00)80999-x

[B42] Saura CA Choi SY Beglopoulos V Malkani S Zhang D Shankaranarayana Rao BS Chattarji S Kelleher RJ 3rd Kandel ER Duff K Loss of presenilin function causes impairments of memory and synaptic plasticity followed by age-dependent neurodegeneration Neuron 2004 42 23 36 15066262 10.1016/s0896-6273(04)00182-5

[B43] Feng R Wang H Wang J Shrom D Zeng X Tsien JZ Forebrain degeneration and ventricle enlargement caused by double knockout of Alzheimer’s presenilin-1 and presenilin-2 Proc Natl Acad Sci USA 2004 101 8162 8167 15148382 10.1073/pnas.0402733101 PMC419574

[B44] Beglopoulos V Sun X Saura CA Lemere CA Kim RD Shen J Reduced beta-amyloid production and increased inflammatory responses in presenilin conditional knock-out mice J Biol Chem 2004 279 46907 46914 15345711 10.1074/jbc.M409544200

[B45] Chen Q Nakajima A Choi SH Xiong X Tang YP Loss of presenilin function causes Alzheimer’s disease-like neurodegeneration in the mouse J Neurosci Res 2008 86 1615 1625 18189321 10.1002/jnr.21601

[B46] Dong S Li C Wu P Tsien JZ Hu Y Environment enrichment rescues the neurodegenerative phenotypes in presenilins-deficient mice Eur J Neurosci 2007 26 101 112 17614943 10.1111/j.1460-9568.2007.05641.x

[B47] Amtul Z Lewis PA Piper S Crook R Baker M Findlay K Singleton A Hogg M Younkin L Younkin SG A presenilin 1 mutation associated with familial frontotemporal dementia inhibits gamma-secretase cleavage of APP and notch Neurobiol Dis 2002 9 269 273 11895378 10.1006/nbdi.2001.0473

[B48] Dermaut B Kumar-Singh S Engelborghs S Theuns J Rademakers R Saerens J Pickut BA Peeters K van den Broeck M Vennekens K A novel presenilin 1 mutation associated with Pick’s disease but not beta-amyloid plaques Ann Neurol 2004 55 617 626 15122701 10.1002/ana.20083

[B49] Raux G Gantier R Thomas-Anterion C Boulliat J Verpillat P Hannequin D Brice A Frebourg T Campion D Dementia with prominent frontotemporal features associated with L113P presenilin 1 mutation Neurology 2000 55 1577 1578 11094121 10.1212/wnl.55.10.1577

[B50] Bai G Chivatakarn O Bonanomi D Lettieri K Franco L Xia C Stein E Ma L Lewcock JW Pfaff SL Presenilin-dependent receptor processing is required for axon guidance Cell 2011 144 106 118 21215373 10.1016/j.cell.2010.11.053 PMC3034090

[B51] Shen J Kelleher RJ III The presenilin hypothesis of Alzheimer’s disease: Evidence for a loss-of-function pathogenic mechanism PNAS 2007 104 403 409 17197420 10.1073/pnas.0608332104 PMC1766397

[B52] Wang R Tang P Wang P Boissy RE Zheng H Regulation of tyrosinase trafficking and processing by presenilins: partial loss of function by familial Alzheimer’s disease mutation Proc Natl Acad Sci USA 2006 103 353 358 16384915 10.1073/pnas.0509822102 PMC1326180

[B53] Kang DE Soriano S Frosch MP Collins T Naruse S Sisodia SS Leibowitz G Levine F Koo EH Presenilin 1 facilitates the constitutive turnover of beta-catenin: differential activity of Alzheimer’s disease-linked PS1 mutants in the beta-catenin-signaling pathway J Neurosci 1999 19 4229 4237 10341227 10.1523/JNEUROSCI.19-11-04229.1999 PMC6782616

[B54] Landman N Jeong SY Shin SY Voronov SV Serban G Kang MS Park MK Di Paolo G Chung S Kim TW Presenilin mutations linked to familial Alzheimer’s disease cause an imbalance in phosphatidylinositol 4,5-bisphosphate metabolism Proc Natl Acad Sci USA 2006 103 19524 19529 17158800 10.1073/pnas.0604954103 PMC1748258

[B55] Tu H Nelson O Bezprozvanny A Wang Z Lee SF Hao YH Serneels L De Strooper B Yu G Bezprozvanny I Presenilins form ER Ca2+ leak channels, a function disrupted by familial Alzheimer’s disease-linked mutations Cell 2006 126 981 993 16959576 10.1016/j.cell.2006.06.059 PMC3241869

[B56] Baki L Neve RL Shao Z Shioi J Georgakopoulos A Robakis NK Wild-type but not FAD mutant presenilin-1 prevents neuronal degeneration by promoting phosphatidylinositol 3-kinase neuroprotective signaling J Neurosci 2008 28 483 490 18184791 10.1523/JNEUROSCI.4067-07.2008 PMC6670519

[B57] Pratt KG Zimmerman EC Cook DG Sullivan JM Presenilin 1 regulates homeostatic synaptic scaling through Akt signaling Nat Neurosci 2011 14 1112 1114 21841774 10.1038/nn.2893 PMC3164917

[B58] Lazarov O Morfini GA Pigino G Gadadhar A Chen X Robinson J Ho H Brady ST Sisodia SS Impairments in fast axonal transport and motor neuron deficits in transgenic mice expressing familial Alzheimer’s disease-linked mutant presenilin 1 J Neurosci 2007 27 7011 7020 17596450 10.1523/JNEUROSCI.4272-06.2007 PMC2801050

[B59] Durkin JT Murthy S Husten EJ Trusko SP Savage MJ Rotella DP Greenberg BD Siman R Rank-order of potencies for inhibition of the secretion of abeta40 and abeta42 suggests that both are generated by a single gamma-secretase J Biol Chem 1999 274 20499 20504 10400678 10.1074/jbc.274.29.20499

[B60] Sato T Dohmae N Qi Y Kakuda N Misonou H Mitsumori R Maruyama H Koo EH Haass C Takio K Potential link between amyloid beta-protein 42 and C-terminal fragment gamma 49-99 of beta-amyloid precursor protein J Biol Chem 2003 278 24294 24301 12707272 10.1074/jbc.M211161200

[B61] Zhang L Song L Terracina G Liu Y Pramanik B Parker E Biochemical characterization of the gamma-secretase activity that produces beta-amyloid peptides Biochemistry 2001 40 5049 5055 11305921 10.1021/bi0028800

[B62] Xu Q Bernardo A Walker D Kanegawa T Mahley RW Huang Y Profile and Regulation of Apolipoprotein E (ApoE) expression in the CNS in mice with targeting of green fluorescent protein gene to the ApoE locus J Neurosci 2006 26 4985 4994 16687490 10.1523/JNEUROSCI.5476-05.2006 PMC6674234

[B63] Xu Q Li Y Cyras C Sanan DA Cordell B Isolation and characterization of apolipoproteins from murine microglia. Identification of a low density lipoprotein-like apolipoprotein j-rich but E-poor spherical particle J Biol Chem 2000 275 31770 31777 10918055 10.1074/jbc.M002796200

[B64] Shi J Zhao CB Vollmer TL Tyry TM Kuniyoshi SM APOE epsilon 4 allele is associated with cognitive impairment in patients with multiple sclerosis Neurology 2008 70 185 190 17460153 10.1212/01.wnl.0000264004.62612.44

[B65] de Frias CM Bunce D Wahlin A Adolfsson R Sleegers K Cruts M Van Broeckhoven C Nilsson LG Cholesterol and triglycerides moderate the effect of apolipoprotein E on memory functioning in older adults J Gerontol B Psychol Sci Soc Sci 2007 62 P112 P118 17379671 10.1093/geronb/62.2.p112

[B66] Mahley RW Weisgraber KH Huang Y Apolipoprotein E4: a causative factor and therapeutic target in neuropathology, including Alzheimer’s disease Proc Natl Acad Sci USA 2006 103 5644 5651 16567625 10.1073/pnas.0600549103 PMC1414631

[B67] Harris FM Brecht WJ Xu Q Tesseur I Kekonius L Wyss-Coray T Fish JD Masliah E Hopkins PC Scearce-Levie K Carboxyl-terminal-truncated apolipoprotein E4 causes Alzheimer’s disease-like neurodegeneration and behavioral deficits in transgenic mice Proc Natl Acad Sci USA 2003 100 10966 10971 12939405 10.1073/pnas.1434398100 PMC196910

[B68] DeMattos RB Cirrito JR Parsadanian M May PC O’Dell MA Taylor JW Harmony JA Aronow BJ Bales KR Paul SM Holtzman DM ApoE and clusterin cooperatively suppress Abeta levels and deposition: evidence that ApoE regulates extracellular Abeta metabolism in vivo Neuron 2004 41 193 202 14741101 10.1016/s0896-6273(03)00850-x

[B69] Kim J Basak JM Holtzman DM The role of apolipoprotein E in Alzheimer’s disease Neuron 2009 63 287 303 19679070 10.1016/j.neuron.2009.06.026 PMC3044446

[B70] Sadowski MJ Pankiewicz J Scholtzova H Mehta PD Prelli F Quartermain D Wisniewski T Blocking the apolipoprotein E/amyloid-beta interaction as a potential therapeutic approach for Alzheimer’s disease Proc Natl Acad Sci USA 2006 103 18787 18792 17116874 10.1073/pnas.0604011103 PMC1654132

[B71] Cao D Lu H Lewis TL Li L Intake of sucrose-sweetened water induces insulin resistance and exacerbates memory deficits and amyloidosis in a transgenic mouse model of Alzheimer disease J Biol Chem 2007 282 36275 36282 17942401 10.1074/jbc.M703561200

[B72] Cramer PE Cirrito JR Wesson DW Lee CY Karlo JC Zinn AE Casali BT Restivo JL Goebel WD James MJ ApoE-directed therapeutics rapidly clear beta-amyloid and reverse deficits in AD mouse models Science 2012 335 1503 1506 22323736 10.1126/science.1217697 PMC3651582

[B73] Holtzman DM In vivo effects of ApoE and clusterin on amyloid-beta metabolism and neuropathology J Mol Neurosci 2004 23 247 254 15181253 10.1385/JMN:23:3:247

[B74] Jiang Q Lee CY Mandrekar S Wilkinson B Cramer P Zelcer N Mann K Lamb B Willson TM Collins JL ApoE promotes the proteolytic degradation of Abeta Neuron 2008 58 681 693 18549781 10.1016/j.neuron.2008.04.010 PMC2493297

[B75] Ye S Huang Y Mullendorff K Dong L Giedt G Meng EC Cohen FE Kuntz ID Weisgraber KH Mahley RW Apolipoprotein (apo) E4 enhances amyloid beta peptide production in cultured neuronal cells: apoE structure as a potential therapeutic target Proc Natl Acad Sci USA 2005 102 18700 18705 16344478 10.1073/pnas.0508693102 PMC1311738

[B76] Wolozin B Cholesterol and the biology of Alzheimer’s disease Neuron 2004 41 7 10 14715130 10.1016/s0896-6273(03)00840-7

[B77] Belinson H Lev D Masliah E Michaelson DM Activation of the amyloid cascade in apolipoprotein E4 transgenic mice induces lysosomal activation and neurodegeneration resulting in marked cognitive deficits J Neurosci 2008 28 4690 4701 18448646 10.1523/JNEUROSCI.5633-07.2008 PMC3844816

[B78] Levi O Dolev I Belinson H Michaelson DM Intraneuronal amyloid-beta plays a role in mediating the synergistic pathological effects of apoE4 and environmental stimulation J Neurochem 2007 103 1031 1040 17666042 10.1111/j.1471-4159.2007.04810.x

[B79] Brecht WJ Harris FM Chang S Tesseur I Yu GQ Xu Q Dee Fish J Wyss-Coray T Buttini M Mucke L Neuron-specific apolipoprotein e4 proteolysis is associated with increased tau phosphorylation in brains of transgenic mice J Neurosci 2004 24 2527 2534 15014128 10.1523/JNEUROSCI.4315-03.2004 PMC6729489

[B80] Cui Y Huang M He Y Zhang S Luo Y Genetic ablation of apolipoprotein A-IV accelerates Alzheimer’s disease pathogenesis in a mouse model Am J Pathol 2011 178 1298 1308 21356380 10.1016/j.ajpath.2010.11.057 PMC3070550

[B81] Selkoe DJ Clearing the brain’s amyloid cobwebs Neuron 2001 32 177 180 11683988 10.1016/s0896-6273(01)00475-5

[B82] Miners JS Baig S Palmer J Palmer LE Kehoe PG Love S Abeta-degrading enzymes in Alzheimer’s disease Brain Pathol 2008 18 240 252 18363935 10.1111/j.1750-3639.2008.00132.x PMC8095507

[B83] Hellstrom-Lindahl E Ravid R Nordberg A Age-dependent decline of neprilysin in Alzheimer’s disease and normal brain: inverse correlation with A beta levels Neurobiol Aging 2008 29 210 221 17098332 10.1016/j.neurobiolaging.2006.10.010

[B84] Hersh LB Rodgers DW Neprilysin and amyloid beta peptide degradation Curr Alzheimer Res 2008 5 225 231 18393807 10.2174/156720508783954703

[B85] Miners JS Van Helmond Z Chalmers K Wilcock G Love S Kehoe PG Decreased expression and activity of neprilysin in Alzheimer disease are associated with cerebral amyloid angiopathy J Neuropathol Exp Neurol 2006 65 1012 1021 17021406 10.1097/01.jnen.0000240463.87886.9a

[B86] Yasojima K Akiyama H McGeer EG McGeer PL Reduced neprilysin in high plaque areas of Alzheimer brain: a possible relationship to deficient degradation of beta-amyloid peptide Neurosci Lett 2001 297 97 100 11121879 10.1016/s0304-3940(00)01675-x

[B87] Chander H Chauhan A Chauhan V Binding of proteases to fibrillar amyloid-beta protein and its inhibition by Congo red J Alzheimers Dis 2007 12 261 269 18057560 10.3233/jad-2007-12308

[B88] El-Amouri SS Zhu H Yu J Marr R Verma IM Kindy MS Neprilysin: an enzyme candidate to slow the progression of Alzheimer’s disease Am J Pathol 2008 172 1342 1354 18403590 10.2353/ajpath.2008.070620 PMC2329843

[B89] Iijima-Ando K Hearn SA Granger L Shenton C Gatt A Chiang H-C Hakker I Zhong Y Iijima K Overexpression of neprilysin reduces Alzheimer amyloid-{beta}42 (A{beta}42)-induced neuron loss and intraneuronal A{beta}42 deposits but causes a reduction in cAMP-responsive element-binding protein-mediated transcription, age-dependent axon pathology, and premature death in drosophila J Biol Chem 2008 283 19066 19076 18463098 10.1074/jbc.M710509200 PMC2441542

[B90] Meilandt WJ Cisse M Ho K Wu T Esposito LA Scearce-Levie K Cheng IH Yu GQ Mucke L Neprilysin overexpression inhibits plaque formation but fails to reduce pathogenic Abeta oligomers and associated cognitive deficits in human amyloid precursor protein transgenic mice J Neurosci 2009 29 1977 1986 19228952 10.1523/JNEUROSCI.2984-08.2009 PMC2768427

[B91] Edbauer D Willem M Lammich S Steiner H Haass C Insulin-degrading enzyme rapidly removes the beta-amyloid precursor protein intracellular domain (AICD) J Biol Chem 2002 277 13389 13393 11809755 10.1074/jbc.M111571200

[B92] Farris W Mansourian S Chang Y Lindsley L Eckman EA Frosch MP Eckman CB Tanzi RE Selkoe DJ Guenette S Insulin-degrading enzyme regulates the levels of insulin, amyloid beta-protein, and the beta-amyloid precursor protein intracellular domain in vivo Proc Natl Acad Sci USA 2003 100 4162 4167 12634421 10.1073/pnas.0230450100 PMC153065

[B93] Ertekin-Taner N Graff-Radford N Younkin LH Eckman C Baker M Adamson J Ronald J Blangero J Hutton M Younkin SG Linkage of plasma Abeta42 to a quantitative locus on chromosome 10 in late-onset Alzheimer’s disease pedigrees Science 2000 290 2303 2304 11125143 10.1126/science.290.5500.2303

[B94] Mueller JC Riemenschneider M Schoepfer-Wendels A Gohlke H Konta L Friedrich P Illig T Laws SM Forstl H Kurz A Weak independent association signals between IDE polymorphisms, Alzheimer’s disease and cognitive measures Neurobiol Aging 2007 28 727 734 16675064 10.1016/j.neurobiolaging.2006.03.009

[B95] Vepsalainen S Parkinson M Helisalmi S Mannermaa A Soininen H Tanzi RE Bertram L Hiltunen M Insulin-degrading enzyme is genetically associated with Alzheimer’s disease in the Finnish population J Med Genet 2007 44 606 608 17496198 10.1136/jmg.2006.048470 PMC2597950

[B96] Cook DG Leverenz JB McMillan PJ Kulstad JJ Ericksen S Roth RA Schellenberg GD Jin LW Kovacina KS Craft S Reduced hippocampal insulin-degrading enzyme in late-onset Alzheimer’s disease is associated with the apolipoprotein E-epsilon4 allele Am J Pathol 2003 162 313 319 12507914 10.1016/s0002-9440(10)63822-9 PMC1851126

[B97] Zhao Z Xiang Z Haroutunian V Buxbaum JD Stetka B Pasinetti GM Insulin degrading enzyme activity selectively decreases in the hippocampal formation of cases at high risk to develop Alzheimer’s disease Neurobiol Aging 2007 28 824 830 16769157 10.1016/j.neurobiolaging.2006.05.001

[B98] Kim M Hersh LB Leissring MA Ingelsson M Matsui T Farris W Lu A Hyman BT Selkoe DJ Bertram L Tanzi RE Decreased catalytic activity of the insulin-degrading enzyme in chromosome 10-linked Alzheimer disease families J Biol Chem 2007 282 7825 7832 17244626 10.1074/jbc.M609168200

[B99] Vepsalainen S Hiltunen M Helisalmi S Wang J van Groen T Tanila H Soininen H Increased expression of Abeta degrading enzyme IDE in the cortex of transgenic mice with Alzheimer’s disease-like neuropathology Neurosci Lett 2008 438 216 220 18455870 10.1016/j.neulet.2008.04.025

[B100] Leal MC Dorfman VB Gamba AF Frangione B Wisniewski T Castano EM Sigurdsson EM Morelli L Plaque-associated overexpression of insulin-degrading enzyme in the cerebral cortex of aged transgenic tg2576 mice with Alzheimer pathology J Neuropathol Exp Neurol 2006 65 976 987 17021402 10.1097/01.jnen.0000235853.70092.ba

[B101] Bernstein HG Ansorge S Riederer P Reiser M Frolich L Bogerts B Insulin-degrading enzyme in the Alzheimer’s disease brain: prominent localization in neurons and senile plaques Neurosci Lett 1999 263 161 164 10213160 10.1016/s0304-3940(99)00135-4

[B102] Zhao L Yao J Mao Z Chen S Wang Y Brinton RD 17beta-Estradiol regulates insulin-degrading enzyme expression via an ERbeta/PI3-K pathway in hippocampus: relevance to Alzheimer’s prevention Neurobiol Aging 2011 32 1949 1963 20053478 10.1016/j.neurobiolaging.2009.12.010 PMC2889185

[B103] Carson JA Turner AJ Beta-amyloid catabolism: roles for neprilysin (NEP) and other metallopeptidases? J Neurochem 2002 81 1 8 12067222 10.1046/j.1471-4159.2002.00855.x

[B104] Llovera RE de Tullio M Alonso LG Leissring MA Kaufman SB Roher AE de Prat Gay G Morelli L Castano EM The catalytic domain of insulin-degrading enzyme forms a denaturant-resistant complex with amyloid {beta} peptide: implications for Alzheimer disease pathogenesis J Biol Chem 2008 283 17039 17048 18411275 10.1074/jbc.M706316200

[B105] Jean L Thomas B Tahiri-Alaoui A Shaw M Vaux DJ Heterologous amyloid seeding: revisiting the role of acetylcholinesterase in Alzheimer’s disease PLoS One 2007 2 e652 17653279 10.1371/journal.pone.0000652 PMC1920558

[B106] Sun MK Alkon DL Links between Alzheimer’s disease and diabetes Drugs Today (Barc) 2006 42 481 489 16894402 10.1358/dot.2006.42.7.973588

[B107] Leibson CL Rocca WA Hanson VA Cha R Kokmen E O’Brien PC Palumbo PJ Risk of dementia among persons with diabetes mellitus: a population-based cohort study Am J Epidemiol 1997 145 301 308 9054233 10.1093/oxfordjournals.aje.a009106

[B108] Ho L Qin W Pompl PN Xiang Z Wang J Zhao Z Peng Y Cambareri G Rocher A Mobbs CV Diet-induced insulin resistance promotes amyloidosis in a transgenic mouse model of Alzheimer’s disease FASEB J 2004 18 902 904 15033922 10.1096/fj.03-0978fje

[B109] Zhao L Teter B Morihara T Lim GP Ambegaokar SS Ubeda OJ Frautschy SA Cole GM Insulin-degrading enzyme as a downstream target of insulin receptor signaling cascade: implications for Alzheimer’s disease intervention J Neurosci 2004 24 11120 11126 15590928 10.1523/JNEUROSCI.2860-04.2004 PMC6730264

[B110] Crouch PJ Harding SM White AR Camakaris J Bush AI Masters CL Mechanisms of Abeta mediated neurodegeneration in Alzheimer’s disease Int J Biochem Cell Biol 2008 40 181 198 17804276 10.1016/j.biocel.2007.07.013

[B111] Moreno H Yu E Pigino G Hernandez AI Kim N Moreira JE Sugimori M Llinas RR Synaptic transmission block by presynaptic injection of oligomeric amyloid beta Proc Natl Acad Sci 2009 106 5901 5906 19304802 10.1073/pnas.0900944106 PMC2659170

[B112] Lee HG Zhu X Castellani RJ Nunomura A Perry G Smith MA Amyloid-beta in Alzheimer disease: the null versus the alternate hypotheses J Pharmacol Exp Ther 2007 321 823 829 17229880 10.1124/jpet.106.114009

[B113] Verkkoniemi A Kalimo H Paetau A Somer M Iwatsubo T Hardy J Haltia M Variant Alzheimer disease with spastic paraparesis: neuropathological phenotype J Neuropathol Exp Neurol 2001 60 483 492 11379823 10.1093/jnen/60.5.483

[B114] Jacobsen JS Wu CC Redwine JM Comery TA Arias R Bowlby M Martone R Morrison JH Pangalos MN Reinhart PH Bloom FE Early-onset behavioral and synaptic deficits in a mouse model of Alzheimer’s disease Proc Natl Acad Sci USA 2006 103 5161 5166 16549764 10.1073/pnas.0600948103 PMC1405622

[B115] Schmitz C Rutten BP Pielen A Schafer S Wirths O Tremp G Czech C Blanchard V Multhaup G Rezaie P Hippocampal neuron loss exceeds amyloid plaque load in a transgenic mouse model of Alzheimer’s disease Am J Pathol 2004 164 1495 1502 15039236 10.1016/S0002-9440(10)63235-X PMC1615337

[B116] LaFerla FM Green KN Oddo S Intracellular amyloid-beta in Alzheimer’s disease Nat Rev Neurosci 2007 8 499 509 17551515 10.1038/nrn2168

[B117] Chui DH Tanahashi H Ozawa K Ikeda S Checler F Ueda O Suzuki H Araki W Inoue H Shirotani K Transgenic mice with Alzheimer presenilin 1 mutations show accelerated neurodegeneration without amyloid plaque formation Nat Med 1999 5 560 564 10229234 10.1038/8438

[B118] Billings LM Oddo S Green KN McGaugh JL LaFerla FM Intraneuronal Abeta causes the onset of early Alzheimer’s disease-related cognitive deficits in transgenic mice Neuron 2005 45 675 688 15748844 10.1016/j.neuron.2005.01.040

[B119] Oddo S Caccamo A Shepherd JD Murphy MP Golde TE Kayed R Metherate R Mattson MP Akbari Y LaFerla FM Triple-transgenic model of Alzheimer’s disease with plaques and tangles: intracellular Abeta and synaptic dysfunction Neuron 2003 39 409 421 12895417 10.1016/s0896-6273(03)00434-3

[B120] Cruz JC Kim D Moy LY Dobbin MM Sun X Bronson RT Tsai LH p25/cyclin-dependent kinase 5 induces production and intraneuronal accumulation of amyloid beta in vivo J Neurosci 2006 26 10536 10541 17035538 10.1523/JNEUROSCI.3133-06.2006 PMC6674706

[B121] Knobloch M Konietzko U Krebs DC Nitsch RM Intracellular Abeta and cognitive deficits precede beta-amyloid deposition in transgenic arcAbeta mice Neurobiol Aging 2007 28 1297 1306 16876915 10.1016/j.neurobiolaging.2006.06.019

[B122] Oakley H Cole SL Logan S Maus E Shao P Craft J Guillozet-Bongaarts A Ohno M Disterhoft J Van Eldik L Intraneuronal beta-Amyloid aggregates, neurodegeneration, and neuron loss in transgenic mice with five familial Alzheimer’s disease mutations: potential factors in amyloid plaque formation J Neurosci 2006 26 10129 10140 17021169 10.1523/JNEUROSCI.1202-06.2006 PMC6674618

[B123] Suo Z Cox AA Bartelli N Rasul I Festoff BW Premont RT Arendash GW GRK5 deficiency leads to early Alzheimer-like pathology and working memory impairment Neurobiol Aging 2007 28 1873 1888 17011668 10.1016/j.neurobiolaging.2006.08.013

[B124] Van Broeck B Vanhoutte G Pirici D Van Dam D Wils H Cuijt I Vennekens K Zabielski M Michalik A Theuns J Intraneuronal amyloid beta and reduced brain volume in a novel APP T714I mouse model for Alzheimer’s disease Neurobiol Aging 2008 29 241 252 17112635 10.1016/j.neurobiolaging.2006.10.016

[B125] D’Andrea MR Nagele RG Wang HY Lee DH Consistent immunohistochemical detection of intracellular beta-amyloid42 in pyramidal neurons of Alzheimer’s disease entorhinal cortex Neurosci Lett 2002 333 163 166 12429373 10.1016/s0304-3940(02)00875-3

[B126] Klyubin I Betts V Welzel AT Blennow K Zetterberg H Wallin A Lemere CA Cullen WK Peng Y Wisniewski T Amyloid beta protein dimer-containing human CSF disrupts synaptic plasticity: prevention by systemic passive immunization J Neurosci 2008 28 4231 4237 18417702 10.1523/JNEUROSCI.5161-07.2008 PMC2685151

[B127] King ME Kan HM Baas PW Erisir A Glabe CG Bloom GS Tau-dependent microtubule disassembly initiated by prefibrillar beta-amyloid J Cell Biol 2006 175 541 546 17101697 10.1083/jcb.200605187 PMC2064590

[B128] Nimmrich V Grimm C Draguhn A Barghorn S Lehmann A Schoemaker H Hillen H Gross G Ebert U Bruehl C Amyloid beta oligomers (A beta(1-42) globulomer) suppress spontaneous synaptic activity by inhibition of P/Q-type calcium currents J Neurosci 2008 28 788 797 18216187 10.1523/JNEUROSCI.4771-07.2008 PMC6671006

[B129] De Felice FG Velasco PT Lambert MP Viola K Fernandez SJ Ferreira ST Klein WL Abeta oligomers induce neuronal oxidative stress through an N-methyl-D-aspartate receptor-dependent mechanism that is blocked by the Alzheimer drug memantine J Biol Chem 2007 282 11590 11601 17308309 10.1074/jbc.M607483200

[B130] Shankar GM Bloodgood BL Townsend M Walsh DM Selkoe DJ Sabatini BL Natural oligomers of the Alzheimer amyloid-beta protein induce reversible synapse loss by modulating an NMDA-type glutamate receptor-dependent signaling pathway J Neurosci 2007 27 2866 2875 17360908 10.1523/JNEUROSCI.4970-06.2007 PMC6672572

[B131] Resende R Moreira PI Proenca T Deshpande A Busciglio J Pereira C Oliveira CR Brain oxidative stress in a triple-transgenic mouse model of Alzheimer disease Free Radic Biol Med 2008 44 2051 2057 18423383 10.1016/j.freeradbiomed.2008.03.012

[B132] Eckerta A Hauptmannb S Scherpingb I Rheina V Müller-Spahna F Götzc J Müllerb W Soluble beta-amyloid leads to mitochondrial defects in amyloid precursor protein and Tau Transgenic mice Neurodegenerative Dis 2008 5 157 159 10.1159/000113689 18322377

[B133] Chimon S Shaibat MA Jones CR Calero DC Aizezi B Ishii Y Evidence of fibril-like beta-sheet structures in a neurotoxic amyloid intermediate of Alzheimer’s beta-amyloid Nat Struct Mol Biol 2007 14 1157 1164 18059284 10.1038/nsmb1345

[B134] Walsh DM Selkoe DJ Deciphering the molecular basis of memory failure in Alzheimer’s disease Neuron 2004 44 181 193 15450169 10.1016/j.neuron.2004.09.010

[B135] Lacor PN Buniel MC Furlow PW Clemente AS Velasco PT Wood M Viola KL Klein WL Abeta oligomer-induced aberrations in synapse composition, shape, and density provide a molecular basis for loss of connectivity in Alzheimer’s disease J Neurosci 2007 27 796 807 17251419 10.1523/JNEUROSCI.3501-06.2007 PMC6672917

[B136] Barry AE Klyubin I Mc Donald JM Mably AJ Farrell MA Scott M Walsh DM Rowan MJ Alzheimer’s disease brain-derived amyloid- -mediated inhibition of LTP in vivo is prevented by immunotargeting cellular prion protein J Neurosci 2011 31 7259 7263 21593310 10.1523/JNEUROSCI.6500-10.2011 PMC6622598

[B137] Alexandru A Jagla W Graubner S Becker A Bauscher C Kohlmann S Sedlmeier R Raber KA Cynis H Ronicke R Selective hippocampal neurodegeneration in transgenic mice expressing small amounts of truncated a is induced by pyroglutamate-a formation J Neurosci 2011 31 12790 12801 21900558 10.1523/JNEUROSCI.1794-11.2011 PMC6623394

[B138] Um JW Nygaard HB Heiss JK Kostylev MA Stagi M Vortmeyer A Wisniewski T Gunther EC Strittmatter SM Alzheimer amyloid-beta oligomer bound to postsynaptic prion protein activates Fyn to impair neurons Nat Neurosci 2012 15 1227 1235 22820466 10.1038/nn.3178 PMC3431439

[B139] Lesne S Koh MT Kotilinek L Kayed R Glabe CG Yang A Gallagher M Ashe KH A specific amyloid-beta protein assembly in the brain impairs memory Nature 2006 440 352 357 16541076 10.1038/nature04533

[B140] Cheng IH Scearce-Levie K Legleiter J Palop JJ Gerstein H Bien-Ly N Puolivali J Lesne S Ashe KH Muchowski PJ Mucke L Accelerating amyloid-beta fibrillization reduces oligomer levels and functional deficits in Alzheimer disease mouse models J Biol Chem 2007 282 23818 23828 17548355 10.1074/jbc.M701078200

[B141] Walsh DM Klyubin I Fadeeva JV Cullen WK Anwyl R Wolfe MS Rowan MJ Selkoe DJ Naturally secreted oligomers of amyloid beta protein potently inhibit hippocampal long-term potentiation in vivo Nature 2002 416 535 539 11932745 10.1038/416535a

[B142] Abramov E Dolev I Fogel H Ciccotosto GD Ruff E Slutsky I Amyloid-β as a positive endogenous regulator of release probability at hippocampal synapses Nat Neurosci 2009 12 1567 1576 19935655 10.1038/nn.2433

[B143] Palop JJ Chin J Roberson ED Wang J Thwin MT Bien-Ly N Yoo J Ho KO Yu GQ Kreitzer A Aberrant excitatory neuronal activity and compensatory remodeling of inhibitory hippocampal circuits in mouse models of Alzheimer’s disease Neuron 2007 55 697 711 17785178 10.1016/j.neuron.2007.07.025 PMC8055171

[B144] Meyer-Luehmann M Spires-Jones TL Prada C Garcia-Alloza M de Calignon A Rozkalne A Koenigsknecht-Talboo J Holtzman DM Bacskai BJ Hyman BT Rapid appearance and local toxicity of amyloid-beta plaques in a mouse model of Alzheimer’s disease Nature 2008 451 720 724 18256671 10.1038/nature06616 PMC3264491

[B145] Simard AR Soulet D Gowing G Julien JP Rivest S Bone marrow-derived microglia play a critical role in restricting senile plaque formation in Alzheimer’s disease Neuron 2006 49 489 502 16476660 10.1016/j.neuron.2006.01.022

[B146] Fiala M Cribbs DH Rosenthal M Bernard G Phagocytosis of amyloid-beta and inflammation: two faces of innate immunity in Alzheimer’s disease J Alzheimers Dis 2007 11 457 463 17656824 10.3233/jad-2007-11406

[B147] Bolmont T Haiss F Eicke D Radde R Mathis CA Klunk WE Kohsaka S Jucker M Calhoun ME Dynamics of the microglial/amyloid interaction indicate a role in plaque maintenance J Neurosci 2008 28 4283 4292 18417708 10.1523/JNEUROSCI.4814-07.2008 PMC3844768

[B148] Medeiros R Prediger RD Passos GF Pandolfo P Duarte FS Franco JL Dafre AL Di Giunta G Figueiredo CP Takahashi RN Connecting TNF-alpha signaling pathways to iNOS expression in a mouse model of Alzheimer’s disease: relevance for the behavioral and synaptic deficits induced by amyloid beta protein J Neurosci 2007 27 5394 5404 17507561 10.1523/JNEUROSCI.5047-06.2007 PMC6672347

[B149] Town T Laouar Y Pittenger C Mori T Szekely CA Tan J Duman RS Flavell RA Blocking TGF-beta-Smad2/3 innate immune signaling mitigates Alzheimer-like pathology Nat Med 2008 14 681 687 18516051 10.1038/nm1781 PMC2649699

[B150] Tesseur I Zou K Esposito L Bard F Berber E Can JV Lin AH Crews L Tremblay P Mathews P Deficiency in neuronal TGF-beta signaling promotes neurodegeneration and Alzheimer’s pathology J Clin Invest 2006 116 3060 3069 17080199 10.1172/JCI27341 PMC1626127

[B151] Caraci F Battaglia G Bruno V Bosco P Carbonaro V Giuffrida ML Drago F Sortino MA Nicoletti F Copani A TGF-β1 pathway as a new target for neuroprotection in Alzheimer’s disease CNS Neurosci Ther 2011 17 237 249 19925479 10.1111/j.1755-5949.2009.00115.x PMC6493850

